# Supplementary material for: Identification of photoperiod-induced specific miRNAs in the adrenal glands of Sunite sheep (Ovis aries)
Source: Front Vet Sci. 2022 Jul 22;9:888207. doi: 10.3389/fvets.2022.888207 (PMC9354845; doi:10.3389/fvets.2022.888207)
Supplement: Supplementary file 1 [file Data_Sheet_1.ZIP › Supplementary file/miRDeep2 file.zip!/miRDeep2 file/Know_Structure/SP42A2/oar-mir-152.pdf]

miRBase precursor : oar-mir-152
Total read count : 22460
oar-miR-152 read count : 22460
remaining reads : 0

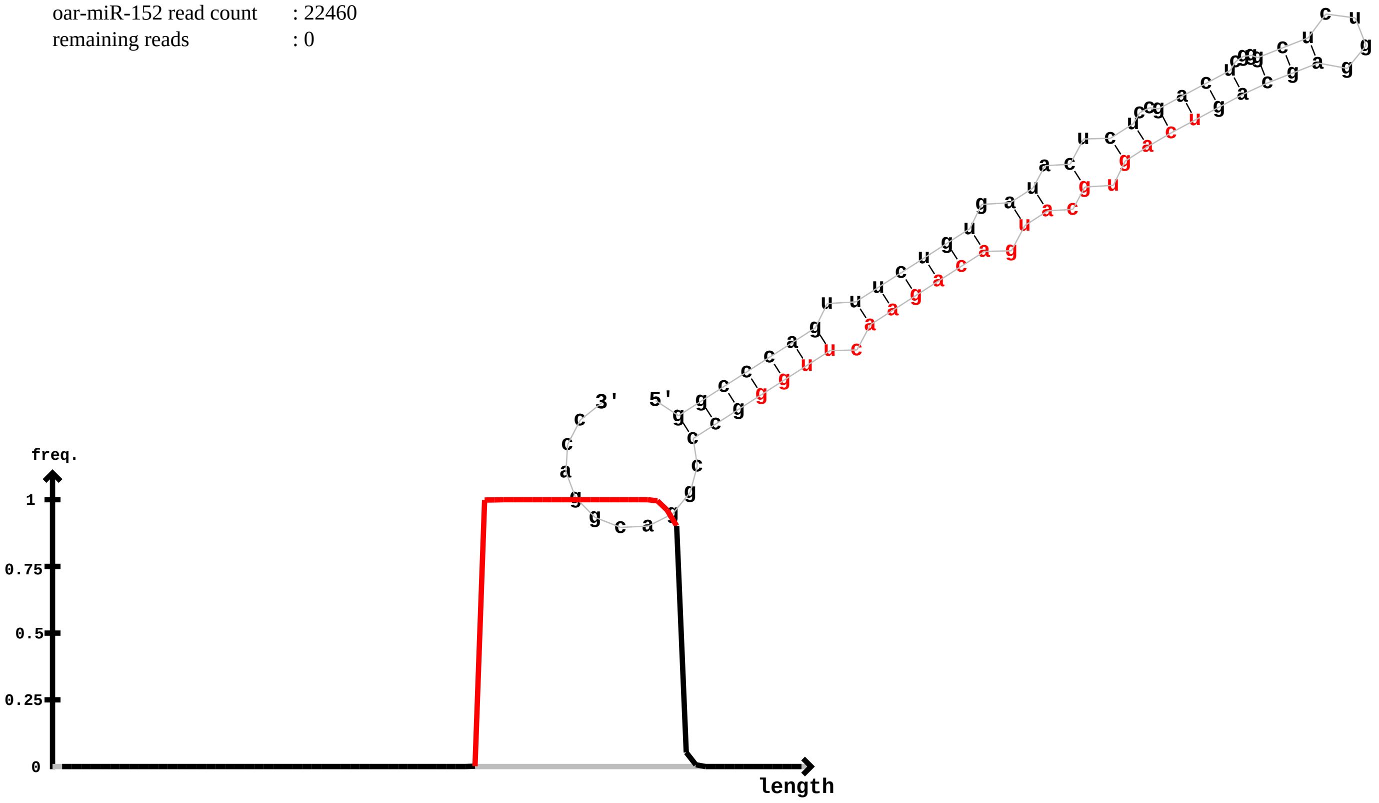

oar-miR-152

```
5'- ggcccaguuucugugauacucuccgacucgggcucuggagcagucagugcaugacagaacuugggcccggacggacc   -3'    exp
    (((((((.((((((.((.(.((..((((...(((....))))))))).).)).)))))).))))))).........    reads      mm         sample
    ..........................................gucagugcaugacagaacuug..............    11         0          seq
    ..........................................gucagugcaugacagaacuugg.............    21         0          seq
    ...........................................ucagugcaugacagaac.................    3          0          seq
    ...........................................ucagugcauCacagaac.................    2          1          seq
    ...........................................ucagugcauCacagaacu................    21         1          seq
    ...........................................ucagugcaugacagaacu................    69         0          seq
    ...........................................ucagugcaugacUgaacu................    1          1          seq
    ...........................................ucagugcauCacagaacuu...............    40         1          seq
    ...........................................ucagugcaugacagaacuu...............    730        0          seq
    ...........................................ucagugcaugacagaacuug..............    1322       0          seq
    ...........................................ucagugAaugacagaacuug..............    1          1          seq
    ...........................................ucagugcaugacagaacuugg.............    19027      0          seq
    ...........................................ucagugcaugacagaacuuggg............    1033       0          seq
    ...........................................ucagugcaugacagaacuugggc...........    149        0          seq
    ............................................cagugcaugacagaacuu...............    2          0          seq
    ............................................cagugcaugacagaacuugg.............    7          0          seq
    ............................................cagugcaugacagaacuuggg............    1          0          seq
    .............................................agugcaugacagaacuu...............    1          0          seq
    .............................................agugcaugacagaacuugg.............    14         0          seq
    .............................................agugcaugacagaacuugA.............    3          1          seq
    .............................................agugcaugacagaacuuggg............    1          0          seq
    .............................................agugcaugacagaacuugggc...........    1          0          seq
```
